# “Are we getting the biometric bioethics right?” – the use of biometrics within the healthcare system in Malawi

**DOI:** 10.1080/11287462.2020.1773063

**Published:** 2020-06-05

**Authors:** Mphatso Mwapasa, Kate Gooding, Moses Kumwenda, Marriott Nliwasa, Kruger Kaswaswa, Rodrick Sambakunsi, Michael Parker, Susan Bull, Nicola Desmond

**Affiliations:** a College of Medicine, University of Malawi, Blantyre, Malawi; b Malawi Liverpool Wellcome Trust Clinical Research Programme (MLW), Blantyre, Malawi; c The Ethox Centre, University of Oxford, Oxford, UK; d Liverpool School of Tropical Medicine, Liverpool, UK

**Keywords:** Biometrics, bioethics, healthcare system, Malawi

## Abstract

Biometrics is the science of establishing the identity of an individual based on their physical attributes. Ethical concerns surrounding the appropriate use of biometrics have been raised, especially in resource-poor settings. A qualitative investigation was conducted to explore biometrics clients (*n *= 14), implementers (*n *= 12) and policy makers as well as bioethicists (*n *= 4) perceptions of the ethical aspects of implementing biometrics within the healthcare system in Malawi. Informed use, privacy and confidentiality as well as perceptions of benefits and harms were identified as major issues in the application of biometrics. Implementation of biometrics within the healthcare system in Malawi poses a range of potential ethical issues and practical challenges that impact on equitable uptake. There is a need for more research to explore the benefits and harms of biometrics in practice. Improved community engagement and sensitization should be a required component of biometrics introduction in Malawi.

## Introduction

Biometrics is defined as the science of establishing and verifying the identity of an individual based on their physical, chemical or behavioural attributes (Jain et al., 2007). Some of the features that are measured in biometrics include DNA, facial features, fingerprints, eye retinas and irises, hand geometry, handwriting, veins and voice (Jain, 2006; Mordini & Massari, 2008).

The use of biometrics to identify patients within the healthcare system comes with a number of potential advantages, such as reducing medical errors, reducing the risk of fraud and improving capacity to react to medical emergencies (Mordini & Ottolini, 2007). Conversely, the use of this technology also comes with a number of challenges. For instance, there is evidence that older respondents are significantly less likely to agree with the introduction of biometrics (Moody, 2004). It has also been revealed that although the use of a fingerprint for authentication may be more easily accepted within financial contexts, acceptability levels in the healthcare service could be low (Jones et al., 2007). Furthermore, the use of biometrics in healthcare systems also raises ethical issues. The main concerns previously identified in the literature relate to data security. Here the concerns raised are not with the use of biometric technologies *per se*, but in how they are applied and how the resulting data is used (Mordini & Massari, 2008; Wickins, 2007). For instance, ensuring that personal health records are appropriately protected from unauthorized use and patient confidentiality is maintained are both considered critical.

The use of biometrics in the Malawian healthcare system is a new concept and the Ministry of Health (MoH) is yet to produce a policy to guide its implementation. Currently, this technology is used at some anti-retroviral therapy (ART) clinics in government health centres with support from non-governmental organizations. Although there is an increase in the use of biometrics within ART clinics in the Malawi healthcare system, the views and concerns of policy makers and communities about biometrics, and in particular on issues that may raise ethical concerns, have not been explored. At this early juncture, it is therefore valuable to explore perspectives and identify potential ethical issues arising from the use of this technology which will lead to an informed policy development. This paper reports on the results of a study that sought to identify views about ethics and best practices regarding the use of biometrics within the Malawian healthcare system.

## Materials and methods

### Study setting

Malawi is a land-locked country located in the southeast of Africa, bordered by Mozambique to the east, south and southwest, Zambia to the west and Tanzania to the north and northeast. It has a total population of about 17 million (8.593 million males and 8.622 females) as of 2015 (DESA U, 2015). The country’s economy is predominantly agricultural, with about 90% of the population living in rural areas. Gross domestic product per capita was 381.37 USD in 2015 (World Bank, 2017).

The country is divided into four major regions: Southern, Central, Eastern and Northern.

**Figure 1.**
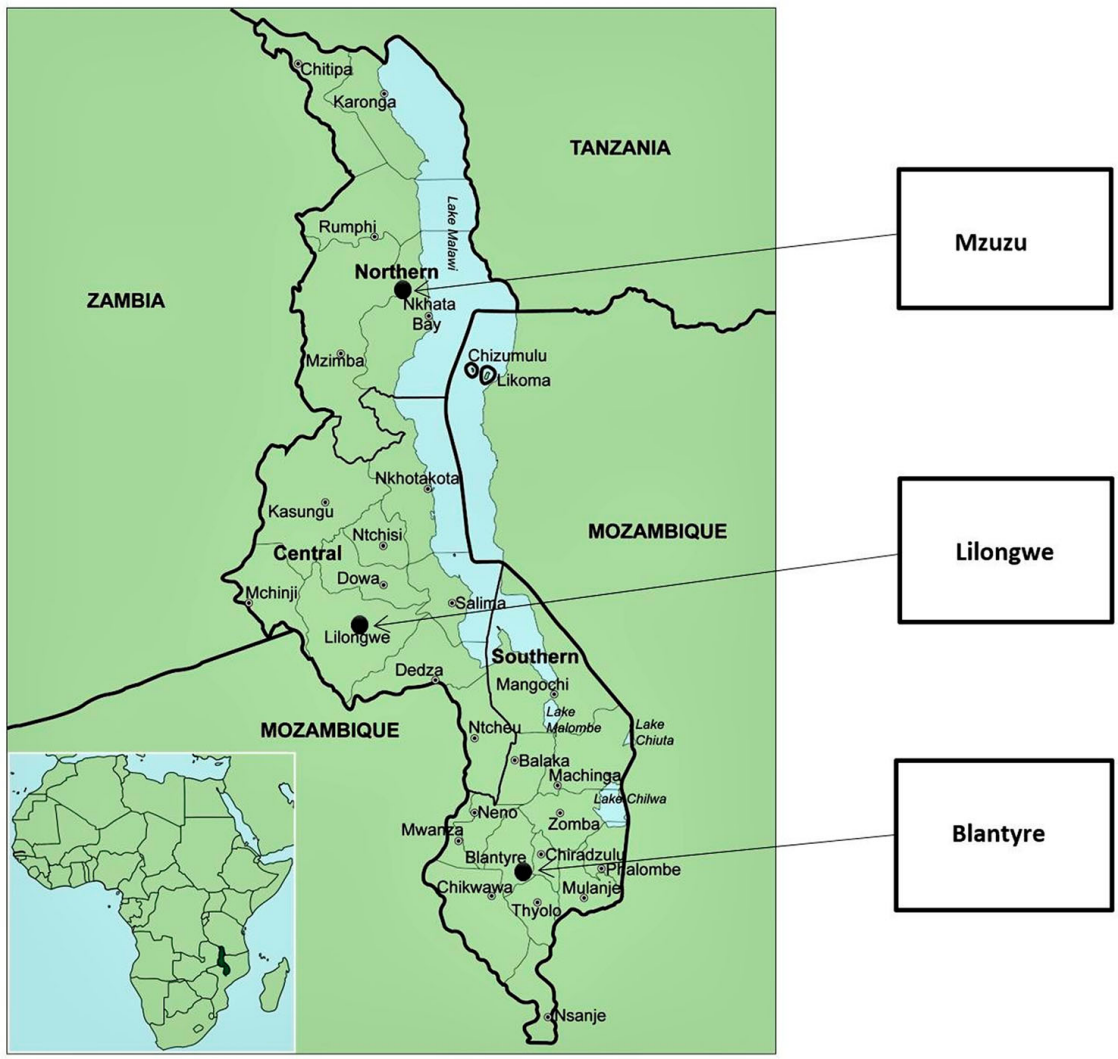
Map of Malawi showing data collection sites. Source of the map: The genus Aloe L. (Asphodelaceae: Alooideae) in Malawi (Klopper et al., 2012).

Study participants were recruited from three districts in Malawi namely: Mzuzu, Lilongwe and Blantyre (see Figure 1). These districts are situated in the Northern, Central and Southern regions of the country. There were no major differences in the health systems among these sites. The only minor difference was that Mzuzu was generally more exposed to the use of biometrics within the healthcare system compared to Lilongwe and Blantyre.

### The healthcare delivery system in Malawi

The Malawi health service delivery system is four-tiered, consisting of community, primary, secondary and tertiary care levels. Community and primary care levels consist of community initiatives, health posts, dispensaries, maternity units, health centres, village health clinics and community and rural hospitals. District and central hospitals provide secondary and tertiary care services, respectively. The former are aimed at treating more specialised conditions while the latter provide referral services for highly specialized conditions (Government of Malawi, 2002; Zere et al., 2007).

Delivery levels within the health system are linked to each other through an elaborate referral system. The use of biometrics within the ART programme is currently confined to clinics situated at secondary or tertiary healthcare levels as these have both human and technical capacity for effective implementation.

Hospital data for this study was collected from three central hospitals and one community hospital, each of which uses a fingerprint as a biometric tool in the registration, tracking, verification and ongoing management of clients at ART clinics.

### Storage of patient information

At present, patient health records are primarily recorded in health booklets, also known as health passports, which were introduced in the late 1990s to improve the quality of personal healthcare. Health passports contain records of the medical history of the individual, assessments of current problems and types of care given. These booklets are sold to clients at a nominal cost of MK 100.00 (≈US$ 0.02) to ensure re-supply of booklets without increasing burdens on overstretched government financial resources (Chaulagai et al., 2005). Health centres are increasingly recording and maintaining electronic health records, but these are not ubiquitous or consistently networked and do not currently track patients between different service levels. Consequently, if patients require healthcare from different providers, they are expected to bring and present their health passport so the clinician is able to follow the client’s clinical history.

### Biometric identification in Malawi

Malawi implemented country-wide registration for a national identification system in 2017. The country is yet to experience the full dividends from the system as it has just started to operate and is not yet linked to the health system. At present, there is still no formal identification and authentication system when patients seek care within the health system. However, biometrics in Malawi is currently used for identification purposes in a few places including National Road Traffic and Safety Services, the electoral commission, the immigration service and banks. However, its use is confined to urban settings and rural populations have little exposure to them. It is also important to note that thumbprints are not just used for biometric identification purposes but are also widely used instead of a signature amongst those with limited literacy, including in medical research.

### Data collection

The study employed qualitative methods of data collection and analysis. Topic guides were developed from a review of the available literature. Deductively developed themes for interviews included biometric policies, perceptions about and experiences of the use of biometrics as well as ethical issues associated with the use of biometrics and suggestions for ways forward.

Purposive sampling was used: the sampling strategy sought to identify key people with different areas of expertise and experiences of biometrics (Coyne, 1997; Marshall, 1996). Potential participants with the knowledge of the implementation of biometrics and policy-making experience on the same were identified during informal interviews with academics from the College of Medicine and hospital directors working in various central hospitals. Biometric clients were drawn from patients seeking ART services because the use of biometrics in the healthcare system is new and currently only piloted in ART clinics at either secondary or tertiary healthcare service delivery. It was noted that some clients were also implementers. In such cases, their responses reflected those of non-client implementers rather than standard clients. Data were collected at policy, health service and community levels using individual semi-structured interviews from February 2015 to January 2016 (see Table 1).

**Table 1.** Categories of research participants and sample size.

Participants	Definition	Male	Female	Total
Biometrics clients	Patients who seek care at the ART (antiretroviral therapy) clinics in health centres using biometrics.	6	8	14
Implementers	Personnel from non-governmental organizations working with the Ministry of Health to implement biometrics. Health Management Information Systems officers at central hospitals.	7	5	12
Policy makers and bioethicists	Personnel at Central Monitoring and Evaluation Department (CMED) of the Ministry of Health	3	1	4

### Data analysis

M.M. conducted and recorded the semi-structured interviews using a digital audio recorder. Interviews were conducted in Chichewa (the vernacular language in Malawi) or English depending on the preference of the interviewee. Audio recordings were later transcribed verbatim in the language in which they were recorded by an experienced transcriber and reviewed for accuracy by M.M. Transcribed data were imported into NVIVO software (Version 10), then coded. Data were coded using both a deductive framework driven by the research design and themes that emerged during inductive content analysis.

Data collection was considered complete when the saturation of information was attained through duplication of emergent findings (Bowen, 2008; Sandelowski, 1995a, 1995b). MM discussed emerging concepts discernible within the coded data with KG before classification of coded data into subthemes and main themes (Coughlin, 2006). Once second-order themes were developed from the original coding framework M.M. and K.G. explored the data again for specific bioethical principles of justice (equity), beneficence and non-maleficence (risks and benefits).

Ethical approval was obtained from the College of Medicine Research Ethics Committee (COMREC) (Approval # P09/14/1622), affiliated to the University of Malawi, and the Liverpool School of Tropical Medicine Ethics Committee (LSTM EC) (Approval # 14.061RS). All study participants provided written consent or thumbprint-witnessed consent after receiving information about the study. We also obtained clearance to collect data at all health centres from the Ministry of Health, Central Monitoring and Evaluation Department.

## Results

Drawing on bioethical principles, three key themes emerged as most prevalent in the data across each of the participant groups. These were informed use, privacy and confidentiality and benefits and harms.

### Informed use

#### Understandings of biometrics amongst ART clients and implementers

Users of novel technologies need to be appropriately informed about them if they are to make informed decisions. There was confusion amongst some patients visiting the ART clinic about why they were supposed to have their fingerprints scanned when they were seeking care at the health centre. Some thought that the use of biometrics is meant for people who are illiterate since fingerprints are usually used in research participants who are illiterate and unable to sign. These sentiments were mostly expressed by biometric clients when they were asked for their views about why the health clinics were migrating from a paper-based system to electronic records with biometric identification.

I am not sure whether you who take fingerprints teach people on why you are doing that because for us we think maybe these people are not able to write and therefore they should just give fingerprint. [Client, Male -BCM_002_150223_002]

Clients did not have enough information to assess the challenges that may come with the use of biometrics. Most of them showed a lack of awareness on the potential applications of biometrics and electronic medical records.

It will be difficult for me to say what disadvantages it could have because every person has this … yes [thumb], but mine [thumb] can’t match with yours, no. Everyone has their own which is unique to them. [Client, Female -BCM001_150930_001]

Both implementers and clients revealed that there was little knowledge of biometrics among their peers even though it is used in some sectors of the healthcare and other official systems. The data also revealed that clients lacked the interest to find out more information on biometrics as all they care about was being treated. This is shown below:

Most clients don’t bother asking about biometrics. All they want is help. We could say ethical guidelines could help address this so that people know why they are being asked to provide finger prints, because they register their fingerprints without knowing why, they do it just because they [are] told about registration. [Implementer, Male – LIN 005_141125_001]

Because we come here ignorantly, and we just have to accept whatever they say because all we want is life. [Client, female -BCM 005_150224_001]

Low levels of understanding of biometrics when used within the ART clinic system were often linked to other factors associated with power imbalances in client–provider relationships which led to a lack of questioning on the part of the client (to the implementer) about the use of fingerprints in such settings. Implementers often attributed this to disinterest on the part of clients. However, both implementers and clients considered that it would be possible to educate their peers about biometrics effectively, if the resources were made available for such sensitization:

For example, when we are [voting], before they take the picture of you, they first of all describe it to you, saying, ‘This is how you are going to do it,’ you find that even the elderly standing in the queue finds it not difficult. So, similarly with this, if we receive training, we Malawians will have had our eyes opened and be able to realise that this thing [biometrics technology] is helpful. [Client & implementer, male – BMC 001_150928_001]

#### Implementers’ understandings of biometrics

In general, implementers and policy makers were able to outline and elaborate on the benefits of the use of biometrics to the healthcare system, unlike clients. This implies that they had a much better understanding of biometrics than the biometrics clients. In addition to potential benefits to patients, they discussed potential benefits to the healthcare system of having networked electronic health records linked to biometric identifiers.

If you look at Malawi, you find out that at the end of the day you have a patient who has six or seven records and four or five health passports. And when you look at all these things, they have an impact on our systems from a public health point of view. You find out that maybe one of the things that you are underestimating is a major issue … So, what basically happens is that if all these things are not properly … documented … you end up having like patients being duplicated, you end up patients who are, like when you are looking at continuity of care, you don’t have like this connectivity between these and it’s very difficult to identify which patient is this. [Implementer, male – BHT 001_141117_001]

In contrast to clients, policy makers and implementers raised a number of concerns about rolling out biometric technologies nationwide in Malawi. Many of these concerns focused on the protection of privacy and confidentiality, as discussed in more detail below.

### Privacy and confidentiality

Clients discussed ways that biometrics could minimize risks related to confidentiality posed by the current paper-based system. While implementers also recognized such benefits, they additionally voiced concerns about potential risks to privacy and confidentiality that may be posed by increasing use of biometrics and electronic health records.

In relation to the potential benefits of biometrics for privacy, clients discussed risks to confidentiality posed by the current use of health passports within the Malawian health system. Clients thought the health passports threatened their privacy and confidentiality because their HIV positive status was clearly indicated within the first few pages of the health passport. A significant number of HIV positive clients consequently preferred to possess two health passports; one that stated their HIV serostatus (which they used when accessing ART treatments), and another one without HIV status (which they could use when accessing other forms of healthcare). Some clients noted that taking such steps to preserve confidentiality and reduce their exposure to stigma as a result of their HIV positive status could adversely affect healthcare provision and that biometrics may, in fact, reduce such impacts without increasing patient exposure to stigma.

What causes people [to have two health passport books] is the desire to hide their HIV status, denial of their condition because they are on medication [ART] … But if you hide the one (Health passport) with details of your HIV status and use the one without, they [doctors] won’t know what your status is like. You may look smart by doing that but ultimately you are hurting yourself. [Client, Female – BCL 001_150225_001]

Most clients felt the use of biometrics linked with an electronic health records system had potential benefits for improving confidentiality and privacy compared to the existing health passport system:

This system would help [a lot] because it means the confidentiality will just be between you and the providers. I feel that’s the advantage the system has, because with my status [referring to HIV positive status], I don’t want everyone to know that that’s my status. [Client, female – BCM 001_150930_001]

Implementers were more likely to identify risks to confidentiality through the use of biometrics. While electronic data records within the healthcare system in Malawi are protected through restricted access to servers and use of passwords, some implementers had doubts as to whether the system was really safe or could be exploited by outsiders.

But I think our system seems weak to some extent and one can attack it and play around with the data of the people and easily escape. So, I am thinking if those responsible could sit down and strengthen the system so that it’s not easily hacked. Because the way the system is at the moment one can get into it and say, He too is on medication [referring to ART]? I did not know. [Implementer, Female – LIN_004_141126_001]

Bioethicists and policy makers also expressed the views that there is a lack of security to protect health and associated biometric data. They additionally expressed concerns that implementing biometric identification and electronic health records could lead to the exploitation of data by western researchers. These concerns relate to broader concerns about increasing storage and sharing of data and biological samples across borders.

It should not be something that should be a technology for data mining and data harvesting, but something that should be used for the benefit of the participants. [Policy maker, Male –- BIO_001_150226_001]

Despite these concerns, there was support amongst many clients, implementers and policy makers for a broader implementation of biometrics in Malawi, provided it could be undertaken in an equitable and sustainable way, as discussed below.

### Benefits and harms

In general terms, implementers and clients considered the use of biometrics coupled with electronic health records to have a great potential to strengthen access to healthcare and improve care by enabling reliable linkage of medical records from different health providers to a single client.

The information you have at one point is the very information you can find at another point. Let us say a patient was registered here and wants to move to Blantyre (another city about 300 kilometres away), the same information will be accessed there … You can easily trace that person unlike when you are using paperwork. [Client & Implementer, Male -BCM 001_150928 _001]

It would be great if our information would appear in the system the moment the system scans our fingers because if I don’t have a health passport book or if I go to another location and run short of drugs, if they scan my finger, it means they will have access to my medical records. [Client, female – BCM 003_150223_003]

Currently, the Malawian healthcare system is overwhelmed with high volumes of patients leading to long queues when it comes to seeking services. However, study participants stated that replacing the use of health passports with biometrics has reduced waiting times in ART clinics where biometrics are being implemented. Both implementers and clients recognized the importance of biometrics in speeding up the flow of patients through the healthcare system as illustrated by two quotes outlined below:

Yes, so if you are to write manually, it becomes a problem. But with the computer is convenient. You are also able to know who visits regularly, with what conditions and what the current condition is immediately. [Client, male & Implementer- BCM_004_150223_004]

Ah, the other advantage is that it’s time effective. Things are processed faster because once you have dipped your finger into the ink and stumped then you are done. But if you are signing, they must demonstrate to you how and where to sign. But with fingerprint, it’s only a matter of seconds and you are done, thereby allowing your friends to also go in and do what you did. [Client, Male_BCM_006_150224_002]

Implementation of biometrics was considered particularly valuable to minimize paperwork. Study participants viewed paperwork as challenging for patients with poor literacy skills. Furthermore, the use of biometrics was favoured by patients compared to paperwork as they felt that it increased equity in access and reduced potential stigma related to illiteracy as one client explained:

Most people in rural settings feel delayed when asked to write. That’s one [reason]. Secondly, sometimes the patient doesn’t know how to write. Therefore, [if biometrics is implemented] there is uniformity in that they are not exposed as illiterate … There will be no difference between the one who went to school and the other who did not go to school. [Client, female – BCM 150929_02]

The use of biometrics is currently restricted to some urban health centres due to wider issues of access to technology with poor rural infrastructure. This raises the issue of whether it is ethical to implement a technology that is likely to benefit urban communities as opposed to rural communities. Views about the importance of equitable access were consistent among biometric clients and implementers as illustrated below:

The challenge that could be there is that I think it’s not implemented in many places yet. Yes, it’s being implemented mostly in town settings … If you go to Nthalire [very remote area] today, I am not going to find it there, it’s like those who benefit the most are those who dwell in towns. [Client, male -BCM_008_150224_004]

We often tend to implement these things just here in town, but we should consider our friends in the villages, because we too came from the villages if we critically look at it. So, if we just think about ourselves, saying, ‘Ah, no, let us just do it here in town’, we are making a mistake … this needs to reach out even to the one residing in the village. [Client & Implementer -BCM _001_150928]

In addition to considering the value of promoting equitable access to biometric identification and electronic healthcare records, implementers discussed the importance of ensuring that any such roll-out was appropriately thought through and sustainably resourced to minimize potential harms. The quote below speaks to the need for such initiatives:

You really need to do research before implementation. Most of the times, here in Malawi, we implement things just because they have been offered to us by donors. A donor comes here and gives you things to do, saying, “Use this,” and off they go without training people, demonstrating to them how it can be used. [Client & Implementer- BMC_150928_001]

Resources for effective implementation of biometrics included a reliable electrical power supply, secured computing equipment (with the capacity to network healthcare records where possible) and access to sufficiently trained IT staff to maintain the systems. Additionally, substantial training for healthcare staff would be required to ensure they know how to use the technology effectively and maintain the confidentiality of data.

Some of the medical personnel are quite of age and they are seeing these things for the first time so they are facing challenges. That’s why I can say no [to whether they have enough knowledge about biometrics] because they have never used a computer before. [Implementer, male – LIN 001_141125_004]

We still haven’t realised the importance of security in everything … They log into a system and just move out of the room without being conscious of having to log out from the system. Therefore, if somebody enters the room and finds the system logged on, they can do whatever they want. [Implementer, male – HOK 001_141127_001]

Implementers considered that further research is needed to determine whether it would be cost-effective to sustainably implement biometric identification and electronic health records more widely within Malawi. Research would also be needed to give some insights towards the development of a policy and governance framework for biometric use which could promote benefits and minimize risks for patients and the health system, as well as an engagement and sensitization strategy to ensure patients and communities were appropriately informed about the new technology.

## Discussion

The study results suggested that widespread implementation of biometric identification would be welcomed and considered ethically appropriate, if undertaken in a manner that ensured that the benefits of implementation for patients and for the healthcare system outweighed potential harms and if such benefits were equitably distributed across geographical regions, gender and literacy levels. Bioethicists, policy makers and implementers expressed heightened awareness of the potential harms arising from the use of the technology. In contrast, clients were unlikely to refer to their right to autonomy, risk of harms and need for justice in the future development of biometrics within the health system. Rather they suggested they would adopt this uncritically. Furthermore, biometrics and electronic health records are perceived to have an advantage over the current health passport system in terms of promoting privacy and confidentiality as well as facilitating effective and timely treatment. Despite this largely positive reception of biometrics, a number of issues were identified that would need to be addressed including the knowledge gap between implementers and clients about the technology; concerns about a lack of security for biometric data and health records; plus potential inequities in the use of biometrics in urban and rural settings. Each of these issues is discussed below.

The knowledge gap that exists among communities about biometrics highlights the importance of raising community and public awareness about the use of biometrics within the healthcare system. The data showed that clients were not informed about the full extent to which the system works or could work. Although most clients did not mind using biometrics, placing themselves as passive recipients as opposed to being active participants in their healthcare system, they were unaware of its potential advantages and disadvantages. However, the knowledge levels of clients who were themselves implementers were much higher than those who were not implementers. This study provides insights to the implementation of biometrics in this setting, and an educational model that can be drawn on to determine how best to inform clients and communities about their implementation.

Although educational attainment at the household level has increased since 1992 (National Statistical Office/Malawi, ICF, 2017), Malawi continues to have high illiteracy levels, particularly among women and people living in rural areas. As a result, effective public and community engagement becomes paramount prior to the implementation of any new health intervention. Experience from the community engagement team at the Malawi Liverpool Wellcome Trust Clinical Research Programme has shown that health information dissemination in Malawi works well when the public is engaged through dialogue, which is rooted in a two-way communication system. In this case, for biometric technology to be comprehensively integrated into the mainstream national healthcare system, there is need for the MoH, in collaboration with other relevant stakeholders to consider developing effective public and community engagement strategies to sensitize the general public, promote uptake and support its use in the health system. Some of the immediate initiatives that can be considered as part of public and community engagement include use of promotional films; development and distribution of information, education and communication (IEC) materials; use of participatory tools such as theatre for development, radio and health exhibitions. In order for these initiatives to be effective, there will be need to diversify the target audience to make sure that no one is left out of the process.

In general, both biometric implementers and clients were accepting of biometric technologies. This differs from Chandra et al. (2008) who found that healthcare providers were more accepting of biometric technologies than consumers. However, it is likely that client responses to biometric technologies are informed by wider differential power relations between implementers and clients and the fact that within health systems in this setting, clients are generally discouraged from asking questions or asserting their rights as service-users.

Fears about biometric data security were commonly expressed among policy makers and implementers. These centred on issues of data security and whether due consideration had been given to the potential risks of data breaches when biometrics systems were put in place. In contrast, clients frequently failed to identify possible risks associated with biometrics, likely because their risk focus was on the health issue for which they were presenting, rather than a more abstract risk of possible future data breaches. This focus on bounded and controllable risk is described by Ulrich Beck as a feature of societies under modernization, rather than those risks that have become incalculable and global with unbounded effects. These latter risks such as those of data security are only recognizable in a risk society, according to Beck, in which processes of modernization have resulted in the production of global risk such as technological or environmental risks (1992).

Due to their greater exposure to this wider global discourse through their exposure to biometric systems, both policy-makers and implementers were more likely to identify and focus on the potential risks associated with the technology. Further and in contrast to patient concerns about privacy and confidentiality of electronic records in some higher-income settings, clients in Malawi considered that electronic records could actually promote privacy and confidentiality in a way that the current system of health passports failed to do. This is likely to be related to the heightened awareness of potential stigma within the research population, a risk that is bounded and calculable since it is driven by proximate rather than global social relations since ART clients had also explained that they often used two health passports to avoid the stigma of being positive when seeking care for other ailments.

Presently, Malawi’s health sector is heavily dependent on foreign resources (Mwansambo, 2015). This scenario creates a challenge for policy makers and implementers because there is a lack of financial, human and infrastructure resources to implement robust security for biometrics and electronic data systems. If biometric technology is to be more broadly implemented within Malawi, it is important that the potential harms of such technologies are minimized for clients and appropriate measures to ensure data security are in place.

There is evidence of long waiting periods for patients in a patient-flow study conducted in three HIV clinics in Uganda (Russell et al., 2016). Similar findings were mirrored by a study conducted by Jafry et al. (2016) on general patients in rural health centres in Malawi. These studies revealed the need to find ways to improve the flow of patients through the health centres. In particular, men in such settings often delay healthcare seeking to avoid spending long hours waiting to be seen (Chikovore et al., 2014). Our findings from ART clinics with established electronic systems show that the use of biometrics and electronic patient record systems may improve the flow of patients through the healthcare system when compared to a paper-based system, thus potentially reducing long queues of patients at health centres. An improved flow of patients within the Malawian health care system could encourage health seeking among male patients who are unwilling to queue for long periods of time and thereby result in an increase in their access to healthcare. Consequently, this may make healthcare more accessible to men and therefore more equitable.

Currently, the use of biometrics and electronic health records within the healthcare system is concentrated in urban centres. Lack of biometrics use in rural health centres may be influenced by a lack of resources to support the technology in such settings. For example, health centres situated in very remote areas are not connected to the national electricity grid. Implementing biometrics in these areas will need alternative sources of electricity such as generators and solar power which may be difficult to maintain due to limited resources. Consequently, although equitable access to biometrics was considered important by multiple stakeholder groups within this study, it may be very challenging to implement biometric technologies nationwide. This raises questions about the appropriateness of allocating scarce resources to an intervention which is more likely to benefit urban than rural populations.

Our study has some limitations. Data were collected from patients in ART clinics and may have limited relevance to other healthcare settings in Malawi. In particular, the benefits of a biometrics system in reducing the stigma associated with health passports indicating HIV positive status are likely to be less relevant amongst HIV negative clients. Furthermore, the results may be relevant to urban settings only as all study participants included in this study were situated in urban health centres. However, the findings are important in stimulating more research on the use of biometrics within the healthcare system to inform policy makers on more ethical approaches to implementing biometrics systems.

## Conclusions

Stakeholders perceive that the implementation of biometrics within a healthcare system in low- and middle-income country settings like Malawi poses a range of potential ethical benefits and risks. There is a need to bring together the perspectives of multiple stakeholders to inform policy making and implementation, and to monitor and evaluate any roll-out of biometrics to assess benefits and harms. While stakeholders in this study discussed the importance of equitable implementation of biometric technologies in both urban and rural settings, cost/benefit evaluations may lead to recommendations that biometric technologies be implemented selectively, depending on the available infrastructure and health needs of specific populations.

If a decision is made to roll-out implementation, priority topics for policy makers and implementers to address in response to stakeholder concerns identified in this study include ensuring that the implementation is appropriately resourced, that data security and the preservation of clients’ privacy and confidentiality is optimized. It is also critical to consider how best to engage with users and communities about the use and implications of biometric identifiers and electronic healthcare records to ensure informed use.
